# Cycloalkane-modified amphiphilic polymers provide direct extraction of membrane proteins for CryoEM analysis

**DOI:** 10.1038/s42003-021-02834-3

**Published:** 2021-11-25

**Authors:** Anna J. Higgins, Alex J. Flynn, Anaïs Marconnet, Laura J. Musgrove, Vincent L. G. Postis, Jonathan D. Lippiat, Chun-wa Chung, Tom Ceska, Manuela Zoonens, Frank Sobott, Stephen P. Muench

**Affiliations:** 1 School of Biomedical Sciences, Faculty of Biological Sciences & Astbury Centre for Structural and Molecular Biology, University of Leeds, Leeds, LS2 9JT UK; 2 Université de Paris, Laboratoire de Biologie Physico-Chimique des Protéines Membranaires, CNRS, UMR 7099, F-75005 Paris, France; 3 Institut de Biologie Physico-Chimique, Fondation Edmond de Rothschild pour le dévelopement de la recherche scientifique, F-75005 Paris, France; 4 Wellcome Centre for Anti-Infectives Research, Drug Discovery Unit, Division of Biological Chemistry and Drug Discovery, University of Dundee, Dundee, DD1 5EH UK; 5 GlaxoSmithKline, Gunnels Wood Road, Stevenage, SG1 2NY UK; 6 UCB Pharma, Slough, SL1 3WE UK; 7 School of Molecular and Cellular Biology, Faculty of Biological Sciences & Astbury Centre for Structural and Molecular Biology, University of Leeds, Leeds, LS2 9JT UK

**Keywords:** Membranes, Cryoelectron microscopy

## Abstract

Membrane proteins are essential for cellular growth, signalling and homeostasis, making up a large proportion of therapeutic targets. However, the necessity for a solubilising agent to extract them from the membrane creates challenges in their structural and functional study. Although amphipols have been very effective for single-particle electron cryo-microscopy (cryoEM) and mass spectrometry, they rely on initial detergent extraction before exchange into the amphipol environment. Therefore, circumventing this pre-requirement would be a big advantage. Here we use an alternative type of amphipol: a cycloalkane-modified amphiphile polymer (CyclAPol) to extract *Escherichia coli* AcrB directly from the membrane and demonstrate that the protein can be isolated in a one-step purification with the resultant cryoEM structure achieving 3.2 Å resolution. Together this work shows that cycloalkane amphipols provide a powerful approach for the study of membrane proteins, allowing native extraction and high-resolution structure determination by cryoEM.

## Abstract (web summary)

Higgins et al. present a cycloalkane-modified amphiphilic polymer that can provide direct extraction of membrane proteins for Cryo-EM analysis. They show its utility by extracting and solving the structure of AcrB to a high resolution of 3.2 Å by single particle cryo-EM.

## Introduction

**Fig. 1:**
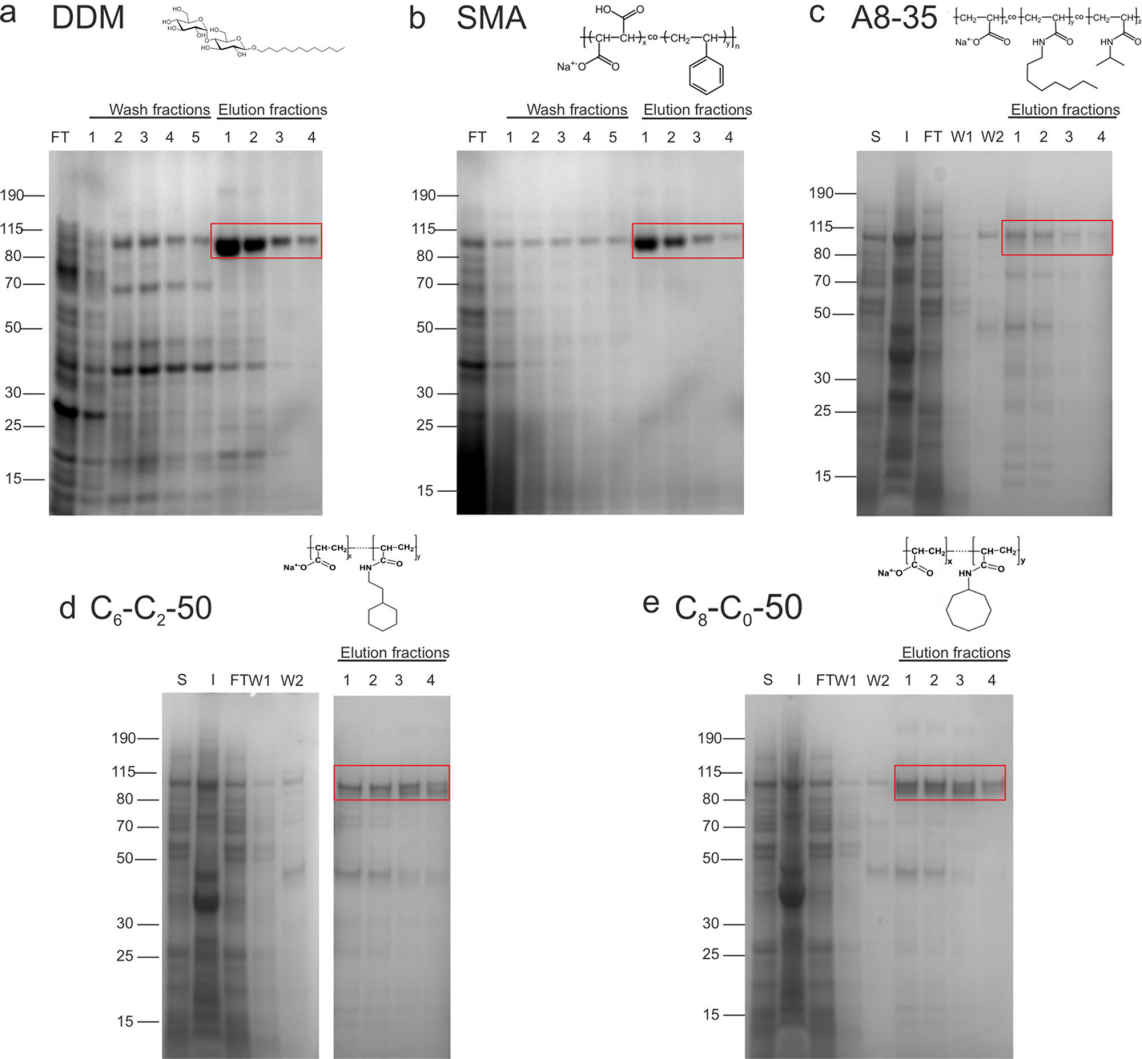
Purification of AcrB in different stabilising systems. SDS-PAGE of purification of AcrB in **a** DDM, **b** SMA, **c** A8-35, **d** C_6_-C_2_-50, and **e** C_8_-C_0_-50. Purification is shown with flow-through (FT) from affinity beads, and wash (W) steps, in addition to soluble (S) and insoluble (I) samples for the amphipol purifications (for experimental details, see the M&M section). All polymers showed the ability to directly extract and purify AcrB with different efficiencies and resulting in different purities. The CyclAPols C_6_-C_2_-50 and C_8_-C_0_-50 showed a reduced efficiency to DDM and SMA (mainly because their concentration used at the solubilisation step is ten times lower) but with much-improved efficiency than the A8-35 polymer.

Membrane proteins represent ~30% of open reading frames in the human genome, and an important class of drug targets^1^ and yet make up only 3% of reported structures in the PDB. Despite their prevalence in the cell and importance for ion transport and cell signalling, amongst other functions, they remain challenging research targets due to problems of overexpression, extraction and stabilisation of their native structure^2–5^. Traditionally extraction and purification of a membrane protein involves the use of a detergent, from which the protein may then be transferred into other surfactants, be they detergents of different chemical composition, protein-based nanodiscs^6^, peptidiscs^7^ or amphipathic polymers^8,9^ (structures for which can be seen in Fig. 1a–e). Extraction of a membrane protein into a detergent micelle functions by disrupting the interaction between protein and its surrounding lipid molecules^10^. Detergent molecules replace the bulk of lipids at the hydrophobic surface of a membrane protein but poorly mimic the lipid bilayer in terms of lateral pressure and thickness which has been shown to cause perturbations in the structure^11,12^. Moreover, the closely associated lipids which can be important for gating, regulation and stability, maybe displaced by competition with the detergent^13–16^. In addition, detergent purification buffers must contain the detergent above its critical micelle concentration (CMC) in all downstream steps which can exacerbate reduction in activity, protein complex dissociation, unnatural oligomerisation and loss of lipid cofactors, amongst other problems^17–19^. Detergent micelles in single-particle cryoEM lead to reduced contrast and increased noise^20,21^ and must be disassembled in native mass spectrometry (MS)^22^. Due to the importance of membrane proteins and the problems associated with detergents, there exist several membrane mimetic alternatives developed to circumvent these. The predominant ones are protein-based nanodiscs^6^ and amphipathic polymers^8,9^.

Classical amphipols (APols) are short and flexible amphipathic polymers able to form complexes with membrane proteins and maintain the proteins in a water-soluble form^8^. They have been used for decades and are well-characterised in their applicability for stabilising membrane proteins^8,23^. The prototypical APol A8-35 is a poly(acrylic acid) (PAA) polymer randomly modified with octylamine and isopropylamine side chains^24^, and many different functionalities have been tethered to the polymer for specific purposes^23,25^. APol A8-35 facilitated the first high-resolution single-particle cryoEM structure of a membrane protein, that of TRPV1^26^. Since then, the number of high-resolution cryoEM structures of membrane proteins using APols (mainly A8-35 and PMAL-C8)^27^ has increased^28^. Of those cryoEM structures deposited within the EMDB, the best resolution achieved using classical APols is 2.17 Å^29^. In addition, APols are amenable to native electrospray ionisation (ESI)-MS^30^. However, A8-35 and the other classical APols traditionally require initial detergent extraction of the protein^31^. Recently, this limitation has been overcome with the development of cycloalkane-modified APols (which contain cyclic rather than linear aliphatic groups) showing much greater efficiency at extracting proteins directly from the membrane than the common A8-35 APol^32^.

The advantage of using polymers for extraction and purification of membrane proteins emerged a decade ago with the use of styrene and maleic acid (SMA) co-polymers in the field of membrane protein research^33^. SMAs heralded the advent of ‘native’ nanodiscs containing a protein directly extracted from the membrane, with its endogenous lipids and without the requirement for conventional detergents^9,34–38^. The styrene-maleic acid-lipid particles (SMALPs) formed^39^ lend themselves to a plethora of biophysical techniques, including cryoEM^40,41^. However, SMALPs also have their limitations (sensitivity to pH extremes and divalent cations), as such there is a continuous interest in developing new SMA-like polymers such as the acrylic acid and styrene polymers (AASTY)^42^ which can be used to directly extract proteins from the membrane, but currently, their applicability to cryoEM has been limited to ~18 Å resolution. Moreover, to date SMA-derived polymers are generally not amenable to ESI MS, having only successfully and recently been applied to native ESI MS for bacteriorhodopsin^43^. Although it has recently been demonstrated that A8-35 can be utilised following protein extraction with SMA^44^, an APol-like polymer combining the extraction capability of SMA with the applications of A8-35 would be highly advantageous.

Here we demonstrate that the properties of A8-35 and SMA can be combined through cycloalkane-modified APols with SMALP-like properties for direct extraction^32^. Using *Escherichia coli* AcrB, we demonstrate that these APol derivatives (henceforth distinguished as CyclAPols) are capable of solubilising the protein of interest directly from the membrane. The CyclAPols can be utilised at exceptionally low concentrations (0.1–0.5%), minimising the risk of destabilisation due to high APol concentrations^45,46^. We present the first cryoEM structure of a protein in CyclAPols, at 3.2 Å resolution, demonstrating their applicability to high-resolution structure determination by cryoEM and making these APols an important new tool in the study of membrane proteins.

## Results

### Cycloalkane-modified amphiphile polymers can solubilise proteins directly from membranes

The CyclAPols (C_6_-C_2_-50 and C_8_-C_0_-50) in addition to A8-35, were compared for their capability for direct membrane solubilisation. *E. coli* membranes overexpressing the exporter AcrB were homogenised and incubated with each polymer before ultracentrifugation to remove insoluble material. Western blot analysis showed that all polymers are capable of solubilising membranes and extracting AcrB, with the amphipathic polymers CyclAPol C_6_-C_2_-50 and C_8_-C_0_-50 showing greater solubilisation efficacy than A8-35 (Fig. S1 and supplementary methods). It was determined that a 0.1% (w/v) concentration of CyclAPols was sufficient for downstream experiments, with yields only slightly lower relative to that obtained for the SMA polymer, despite the lower polymer concentration for CyclAPols (0.1 vs 1%).

A one-step purification with affinity resin was carried out of AcrB extracted with SMA, DDM, A8-35 or CyclAPol (Fig. 1a–e). Under the experimental conditions used, C_8_-C_0_-50 appeared to perform better than C_6_-C_2_-50. This one-step purification procedure with SMA has previously been observed to result in clean homogenous protein^44,47^, with increased purity of SMA-solubilised AcrB relative to detergent^44^. While minor modifications were made to optimise buffers for compatibility with the polymers, purification with CyclAPols resulted in clear elution fractions containing relatively pure AcrB protein consistent with a one-step purification (Fig. 1). The resultant elution fractions of each purification were pooled and dialysed to remove imidazole and protein concentrated to ~1 mg/mL.

**Fig. 2:**
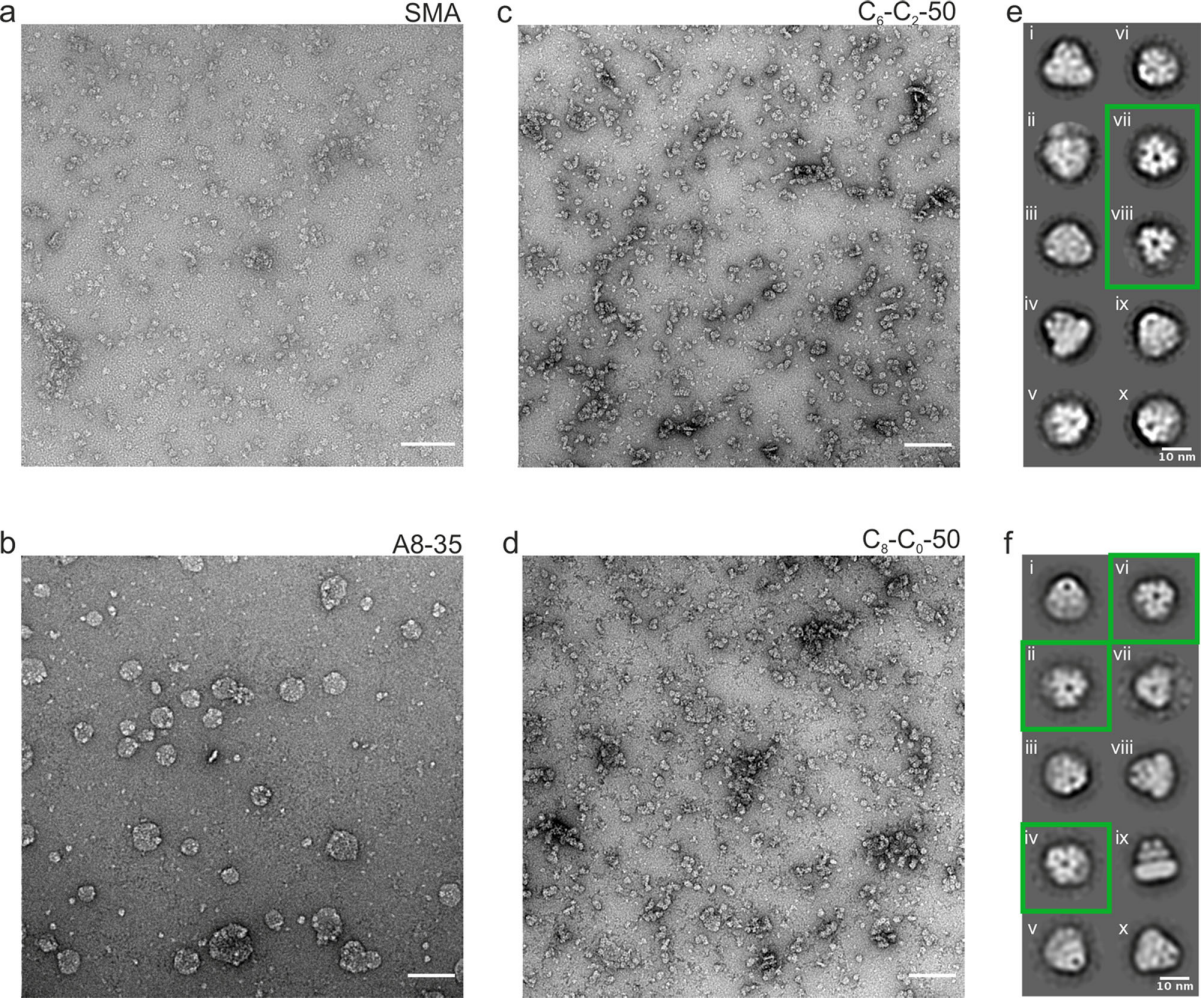
Negative stain electron microscopy of AcrB extracted and purified in SMA, A8-35 and CyclAPols. Representative micrographs were collected at 49k magnification of AcrB following purification in (**a**) SMA, (**b**) A8-35, (**c**) C_6_-C_2_-50, and (**d**) C_8_-C_0_-50. Scale bar is 100 nm in all images. Negative stain 2D classes of AcrB in (**e**) C_6_-C_2_-50 and (**f**) C_8_-C_0_-50 are also shown with some representative high-angle classes highlighted in green. Images (**a)** and (**b**-**d)** were taken using Tecnai F20 and G2-spirit T12 transmission electron microscopes, respectively.

Negative stain electron microscopy was used to assess the homogeneity and stability of AcrB trimer extracted and purified in CyclAPols C_6_-C_2_-50 and C_8_-C_0_-50 showing homogenous, monodisperse protein, with less background contamination than typically observed for detergent micelles and similar to SMA-purified AcrB (Fig. 2 a, c, d). The low level of aggregation, clear trimers and low background observed in the negative stain data for the C_6_-C_2_-50, C_8_-C_0_-50 and SMA samples were indicative of a sample suitable for cryoEM. However, images of A8-35-purified AcrB showed large aggregates which likely contain several copies of AcrB and only a small percentage of monodispersed AcrB (Fig. 2b). The large aggregates suggest that A8-35 at similar concentrations than the other polymers (0.5 vs 0.1% for CyclApols and 1% SMA) is not as efficient as CyclAPols at breaking apart the membrane and may produce larger lipid bilayer fragments containing multiple copies of AcrB. 2D classification of AcrB purified with C_6_-C_2_-50 (e) and C_8_-C_0_-50 (f) showed typical features to those seen with AcrB-SMA^48^ along with increased high angle views in addition to the typical side and top views, particularly for C_8_-C_0_-50 (f, green boxes).

### Single-particle cryoEM of AcrB in CyclAPol C_8_-C_0_-50

We next investigated if CyclAPols, like the classic APols such as A8-35 and PMAL-C8, were also capable of providing a suitable environment for high-resolution structure determination by cryoEM. Purified AcrB was vitrified on Quantifoil grids for single-particle cryoEM analysis. While AcrB extracted and purified in A8-35 was not suitable for cryoEM due to particle aggregation, in the screening of grids both CyclAPols exhibited sufficient particle distribution. AcrB in C_8_-C_0_-50 showed the best distribution and was taken forward for data collection. Consistently, the C_8_-C_0_-50 polymer marginally outperformed C_6_-C_2_-50, with slightly increased purity, yield (Fig. 1) and particle homogeneity as seen in negative stain (Fig. 2) and screening in cryoEM (Fig. S2).

**Fig. 3:**
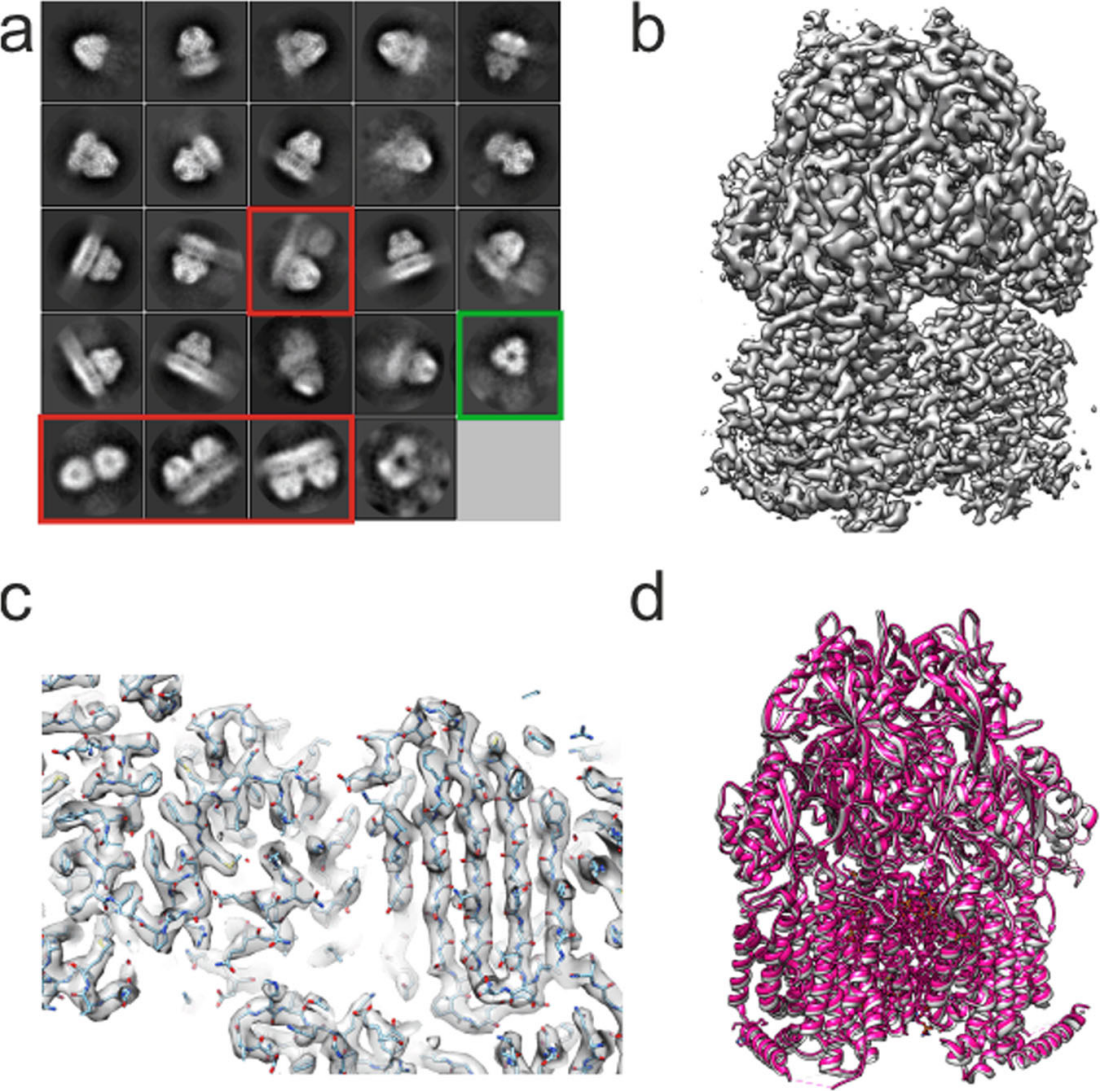
CryoEM of AcrB extracted and purified in CyclAPol C_8_-C_0_-50. **a** Classes of AcrB in C_8_-C_0_-50 following one round of 2D classification. The classes demonstrate a range of views such as a high angle view showing putative threefold symmetry (highlighted in green) with classes showing clear doublets highlighted in red. **b** Side view of the AcrB cryoEM map at 3.2 Å final resolution. **c** Representative map density of AcrB in C_8_-C_0_-50 around the top of the vestibule region. **d** Overlay of the cryoEM structure of AcrB in SMA^40^ (PDB accession code: 6baj; pink) with the AcrB in C_8_-C_0_-50 structure (PDB accession code 7B5P; grey) demonstrating the close similarity of structures.

Following data collection, particle picking was carried out with CrYOLO^49^, and extraction and further processing were carried out in RELION^50^. Approximately 400k particles were initially extracted from 1837 micrographs. Following two rounds of 2D classification, ~200k particles were selected for further 3D classification and processing. Initial 2D classes showed a clear AcrB trimer, with a good angular distribution within the data (Fig. 3a). This is important in reducing the anisotropy in the data that can arise through preferred orientations resulting in reduced map quality. It was noted that a small population of the 2D classes exhibited clear doublets of AcrB trimers (0.5–1% of particles) which had previously been seen in negative stain studies of AcrB in SMA^48^, but not reported in the published structures^40,41^.

**Fig. 4:**
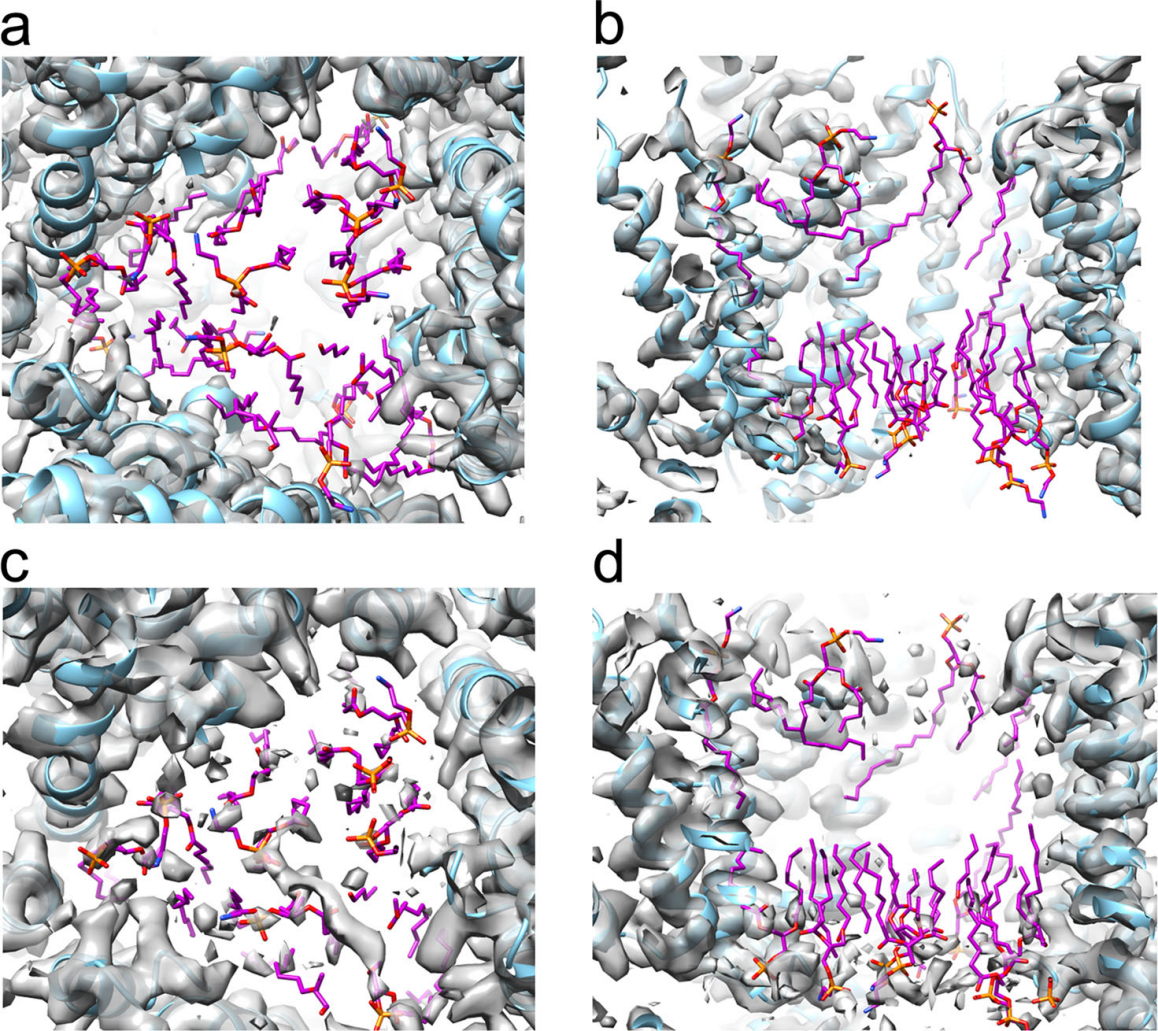
Analysis of the lipid-binding site in AcrB. Density map of C_8_-C_0_-50-purified AcrB seen around the transmembrane region from the side and base. The lipids from AcrB solved by cryoEM in SMA are superimposed and shown in purple, red and orange for carbon, oxygen and phosphate, respectively. The density contour level in (**a**) and (**b**) is comparable to that of Fig. 3c (0.025), a lower threshold of density (0.016) is shown in (**c**) and (**d**) with increased noise. At both contour levels, no density is present that could be assigned to the bound lipids.

The resultant 3D reconstruction, processed with C1 symmetry, achieved a final global resolution of 3.2 Å with clearly resolved density for the secondary structure and in most cases the side chains (Fig. 3, b, c). The local resolution is lower for those helices on the surface of AcrB within the membrane domain, where density for the side chains could not be unambiguously resolved. The previously derived EM structure of AcrB in SMA^40^ was used as a starting point for model building and refinement, with the resultant model being highly similar to previously published AcrB structures^40,41,51^ (Fig. 3d). The structure is asymmetric and exhibits a clear cavity at the interior of the trimer, which after model fitting was devoid of any density that could be assigned to lipids (Fig. 4a–d). This is especially apparent when viewed from the base of the structure, where the trimeric pseudo-symmetry and resolved helices are very clear. Particularly, the structure appears well resolved at the transmembrane region with clear density for the side chains, consistent with a resolution of ~3.5 Å.

### Comparison to AcrB in other amphipathic environments

Comparing the refined structure, especially the chain C of the AcrB trimer, in C_8_-C_0_-50 to the previously published structures in SMA (6baj)^40^ and saposin (6sgu)^51^ using Chimera showed a root mean square deviation (RMSD) of the backbone atoms of 0.7 and 1.5 Å, respectively, reflecting their close similarity. Comparison of the maps (Fig. S3) or overlay of the SMA and CyclAPol structures (Fig. 3d) demonstrates no noticeable difference between structures and only minor variation in loop regions. It is noted that in the reported cryoEM structures of AcrB in SMA or saposin at comparable resolutions, lipids have been identified throughout the transmembrane region. The absence of density observed for lipids in the C_8_-C_0_-50 reconstruction (Fig. 4) leads us to investigate the presence of co-purified lipids in the samples by thin-layer chromatography (Fig. S4 and Supplementary methods). This experiment demonstrates the clear presence of lipids in all samples of AcrB solubilised and purified with the different polymers including C_8_-C_0_-50. Therefore, the apparent lack of lipids in the EM structure is likely due to their mobility which prevents them from being resolved.

## Discussion

Membrane proteins present significant challenges, not least in finding a suitable amphipathic environment that can directly extract the protein from the membrane and stabilise it in an aqueous solution. Although classical APols such as A8-35 are effective in cryoEM, their typical reliance on detergents in the early stages of membrane extraction may be problematic. Using AcrB, we demonstrate that A8-35, as long suspected^8^, may directly extract proteins from the membrane. However, the yield of AcrB extracted with A8-35 is low, confirming the poor detergency property of A8-35 as previously reported. Solubilisation with A8-35 is also incomplete as large objects similar to small vesicles are observed by negative stain EM (Fig. 2), the size of which perhaps could be fine-tuned by A8-35 concentration (currently 0.5%). Although this makes direct extraction of AcrB with A8-35 unsuitable for single-particle cryoEM, the ability to fragment the membrane into larger rafts may be useful for other techniques, such as AFM or mass spectrometry^52^, but was beyond the scope of this study. In contrast to A8-35, the two CyclAPols tested are very effective at solubilising AcrB from the membrane at low concentrations (at an estimated total protein/polymer ratio of 1:1 w/w). This is consistent with the previous finding that the CyclAPols are more efficient than A8-35 at extracting proteins from the membrane, regardless of the target protein^32^. Furthermore, CyclAPol-extracted AcrB (a His-tagged version) can be obtained at a satisfactory level of purity after an affinity purification step. On-going work shows that other affinity tags fused to different bacterial or mammalian membrane proteins including a bacterial ABC transporter (a Strep-tag version) can be also utilised for purification following solubilisation with CyclAPols (Fig. S5).

Importantly, the CyclAPols are compatible with high-resolution cryoEM studies with the resultant 3.2 Å resolution structure of AcrB obtained in CyclAPol C_8_-C_0_-50 being in line with the resolutions regularly obtained with classical APols. There is no noticeable change in structure compared to other amphipathic environments, although lipids are not currently visible, and this represents the joint highest resolution AcrB cryoEM structure^40,51^. We noted a clear improvement in resolution compared to our in-house AcrB-SMA cryoEM reconstructions, the highest resolution of which is ~4.0 Å^41^ and for which the data acquisition setup and data processing pipelines were comparable.

In this study, the CyclAPol C_6_-C_2_-50 appeared less amenable to purification than C_8_-C_0_-50, and while two data collections were attempted with this polymer, the best resolution obtained was 4.4 Å (Fig. S2). As previously noted, while less doublets are visible in 2D classification, a higher proportion of the protein appears aggregated or in multimeric chains after purification with this polymer contributing to a highly diffuse transmembrane region (Fig. S2). This highlights how subtle differences in the chemistry between the two CyclAPols can have an effect on the downstream applications, but this effect may be protein-dependent. The overall architecture of AcrB is near indistinguishable between reconstructions in C_6_-C_2_-50 and C_8_-C_0_-50, although at the lower resolution we may not observe subtle differences.

AcrB is an ideal model protein for such studies as it has been widely characterised with high-resolution cryoEM structures being determined in SMA (3.2 Å),^40^ saposin (3.2 Å)^51^ and most recently in liposomes (3.9 Å)^53^. Studies in saposin^51^ and liposomes^53^ involve reconstitution subsequent to detergent purification. Unlike structures determined in both saposin and SMA, AcrB within liposomes does not appear to show closely associated internal lipids. There were also no identifiable lipids in the cryoEM structure of CyclAPol-purified AcrB, despite the clear evidence of co-purified lipids as observed by TLC experiments (Fig. S4). Although lipids have been detected in other cryoEM studies of AcrB^40,50^^,^ it is possible these are not critical for the function, as AcrZ^51^ or AcrA^54^ may mediate interactions between AcrB and lipids, and structures that show no difference simply reflect increased mobility of the lipid within the AcrB system. Furthermore, in contrast to studies in liposomes, in which a great deal of optimisation of cryoEM conditions was required,^53^ the cryoEM structure obtained here in C_8_-C_0_-50 was the result of a single batch of cryoEM grids with no subsequent optimisation.

The CyclAPols represent an important tool in the field of membrane protein structural studies, which may come to represent an important alternative to detergent and SMA. They extract proteins directly from the membrane at low concentrations and provide a clean purification of the target membrane protein. Compared to the AASTY^42^ polymer, the improved compatibility of CyclAPols with cryoEM shows that changing the chemistry of the polymers can have beneficial effects on sample quality. Moreover, our initial studies have shown that such samples can now be analysed by native MS with the facile release of intact membrane protein complexes for structural analysis with further studies ongoing. We present here the first cryoEM foray into these cycloalkane-modified amphiphile polymer derivatives, anticipating wider applicability to membrane proteins still to be discovered.

Membrane proteins offer great challenges in their study, with a major limitation being in using a solubilising agent that can both solubilise and stabilise the protein of interest and also be applicable to a range of downstream analysis techniques. APols have a strong track record in their applicability to single-particle cryoEM and native MS but have relied on initial detergent extraction which brings with it some limitations. Here we have shown that a modified cycloalkane APol negates the need for initial detergent extraction whilst maintaining the applicability to high-resolution EM structures. This new generation of APols may provide an important addition to the membrane protein toolkit creating more opportunities for membrane protein studies.

## Methods

### Polymer synthesis

Polymers C_6_-C_2_-50 and C_8_-C_0_-50 were synthesised and characterised as described fully in Marconnet et al.^32^ by the grafting of hydrophobic side chains onto polyacrylic acid in the presence of dicyclohexylcarbodiimide. In addition, we used the commercial amphipol A8-35 and DDM from Anatrace. SMA (2:1) supplied unhydrolyzed from Cray Valley which was prepared as previously described in ref. ^36^. Briefly, the unhydrolyzed polymer is solubilised in 1 M NaOH and heated with reflux for 2 h. Once cooled the polymer is precipitated, centrifuged and washed by multiple rounds of resuspension and centrifugation, first in dH2O, then 0.6 M NaOH pH 8, before being freeze-dried and stored.

### Preparation of *E. coli* membranes

*E. coli* membranes were prepared according to chapter 3.4 of Postis et al.^55^. Briefly, the C43(DE3), pRARE2, ΔacrB strain of *E. coli* was used for overexpression by auto-induction in SB media. Cells were harvested, resuspended for cell disruption, cell debris centrifuged and discarded before centrifugation at 100,000×*g* for 2 h to collect the membranes. Membranes were resuspended in a minimal volume (~8 mL) of buffer (50 mM Tris-HCl pH 8.0, 500 mM NaCl, 10% (v/v) glycerol) and frozen for storage at −80 °C. The total protein concentration of the resuspended membranes was measured using a BCA assay as per the manufacturer’s recommendations (Thermo Scientific).

### AcrB purification

Purification of AcrB in SMA was carried out as described previously^44,47^ but with 1% (w/v) SMA at the solubilisation step (see below). For purification in SMA, the solubilisation buffer was 50 mM Tris-HCl pH 8.0, 500 mM NaCl, 10% glycerol. Wash and elution buffers additionally contain 20 mM imidazole and 300 mM imidazole, respectively. Purification in amphipols was similar but buffers were modified to reduce the ionic strength. For this, the solubilisation buffer contained 20 mM Tris-HCl pH 8.0, 250 mM NaCl, 5% glycerol. Wash and elution buffers were supplemented with 10 mM imidazole and 300 mM imidazole, respectively. A second purification was also carried out, which provided the sample of AcrB in CyclAPol C_6_-C_2_-50 for cryoEM data collections. For this second purification, the protocol was largely similar but an extra resin wash was carried out with buffer containing 50 mM imidazole, and the elution was performed with 500 mM imidazole.

Purification was carried out with membranes homogenised in solubilisation buffer to 1 mg/mL. Typically, 1 g of the membrane (at protein concentration 25 mg/mL), was homogenised in 25 mL. SMA at 1% (w/v) was added directly from powder, while CyclAPols (0.1%) and A8-35 (0.5%) were added from 5% (w/v) stock solutions and samples were incubated for 2 h at room temperature (25 ˚C) before ultracentrifugation at 100,000×*g* for 1 h at 4 °C to remove insoluble material.

For each gram of solubilised membrane, 1 mL of final cobalt resin was used. HisPur Cobalt Slurry (Thermo) was prepared by 3x washes each with ddH2O, solubilisation buffer and wash buffer (containing 10 mM imidazole). The soluble material was incubated with this equilibrated cobalt resin overnight at 4 °C with rotation. The flow-through was collected, the resin washed with 5 column volumes (5 mL) solubilisation buffer and 5 column volumes wash buffer before elution fractions were collected and analysed by SDS-PAGE. Elution fractions containing purified AcrB were pooled, placed inside SnakeSkin 10 kDa MWCO dialysis tubing (ThermoFisher) and dialysed overnight against 300 mL solubilisation buffer (20 mM Tris-HCl pH 8.0, 250 mM NaCl, 5% glycerol) at 4 °C, with three buffer changes to remove imidazole. Samples were then concentrated using 100 kDa MWCO nitrocellulose concentrator (Merck) and the final concentration was measured using a DS-11 Spectrophotometer (DeNovix). Final samples for A8-35 and CyclAPols were in 20 mM Tris-HCl pH 8.0, 250 mM NaCl, 5% glycerol.

### Negative stain electron microscopy

Purified AcrB was diluted to 50 μg/mL in solubilisation buffer. Three microlitres of the sample was applied to a glow-discharged carbon grid, incubated for 30 s and excess removed with blotting paper. The grid was washed with double-distilled water and stained with 1% uranyl acetate. Grids of APol-purified AcrB were imaged at 50k magnification using a Tecnai G2-spirit T12 transmission electron microscope (FEI) fitted with a 120 keV Lab6 electron source and Ultra Scan 4000 CCD camera (Gatan). Grids of SMA-purified AcrB were imaged using a Tecnai F20 transmission electron microscope (FEI) fitted with a 200 keV FEG electron source and a CETA CMOS CCD camera (FEI).

### Cryo-electron microscopy

Quantifoil 1.2/1.3 cryo-electron microscopy (cryoEM) grids (QUANTIFOILS) were prepared by glow discharging with a 208-carbon High Vacuum Carbon Coater (Cressington). Purified AcrB at ~1 mg/mL in 20 mM Tris-HCl pH 8.0, 250 mM NaCl, 5% glycerol, after solubilisation with 0.5% A8-35, 0.1% C_6_-C_2_-50 and 0.1% C_8_-C_0_-50 was applied to grids. CryoEM specimens were prepared with a FEI Vitrobot grid preparation robot at 4 °C and 100% humidity by applying 3 μl of the sample (~1 mg/mL) to glow-discharged grids, blotting for 6 s with a blot force of 6 before freezing in liquid ethane. Grids were stored in liquid nitrogen and imaged subsequently using a Titan Krios G3i cryo transmission electron microscope (FEI) at 300 keV voltage equipped with a Gatan K2 Summit camera at the Astbury Biostructure Laboratory. Grids were screened to assess ice thickness, AcrB concentration, monodispersion and homogeneity.

### Electron microscopy data acquisition

Movies were acquired in electron counting mode with a pixel size of 1.07 Å, an exposure rate of 6.6 electrons per pixel per second, and a total exposure time of 10 s divided in 40 frames. Frame alignment and exposure weighting were performed with Motioncor^56^. Contrast transfer function parameters were estimated from the exposure-weighted averages of movie frames with CTFFIND^57^.

### Image processing

Automated picking of particles was carried out using crYOLO^50^ with the general model trained on a subset of particles and picking threshold at 0.2. From 1837 micrographs 409,113 particles were picked of which 402,672 were extracted into RELION ^51^. Two rounds of 2D classification and three rounds of 3D classification were carried out, reducing particle numbers to 100k, prior to further refinement. The map for AcrB structure in SMA^40^, EMD-7074, in a 256-pixel box and low-pass filtered to 30 Å was used as an initial model. The dataset was also processed in cryoSPARC^58^, from the raw image stage, obtaining a similar resolution of 3.3 Å at the final stage of refinement. As cryoSPARC’s own algorithms were used for automated picking and model generation this served as an internal control that no bias was imposed. The model was produced by the manual fitting of the AcrB structure obtained in SMA (6baj), with lipids removed, into the map. One round of real-space refinement in Phenix was performed before fitting in Coot. Sidechains were deleted where unambiguous density was not observed. The construct used possesses two additional N-terminal residues and a C-terminus extension including a His-tag. However, these were not seen in the final map, and numbering was matched to the canonical *E. coli* sequence. Data collection and processing statistics are given in Table 1.

**Table 1:** Data collection and processing statistics for AcrB in a cycloalkane-modified amphiphilic polymer.

	AcrB (EMDB-12043) (PDB 7B5P)
Data collection and processing	
Magnification	130,000
Voltage (kV)	300
Electron exposure (e–/Å^2^)	58
Defocus range (μm)	1.7–3.5
Pixel size (Å)	1.07
Symmetry imposed	C1
Initial particle images (no.)	40,2672
Final particle images (no.)	10,0932
Map resolution (Å)	3.2
FSC threshold	0.143
Map resolution range (Å)	3.19–6.12
Refinement	
Initial model used (PDB code)	6baj
Model resolution (Å)	3.3
FSC threshold	0.5
Model resolution range (Å)	
Map sharpening *B* factor (Å^2^)	−75
Model composition	
Non-hydrogen atoms	22527
Protein residues	3153
Ligands	0
*B* factors (Å^2^)	
Protein	46
Ligand	
R.m.s. deviations	
Bond lengths (Å)	0.009
Bond angles (°)	0.997
Validation	
MolProbity score	2.71
Clashscore	25
Poor rotamers (%)	2.27
Ramachandran plot	
Favoured (%)	90
Allowed (%)	9
Disallowed (%)	1

CryoEM data collection, refinement and validation statistics

### Reporting Summary

Further information on research design is available in the Nature Research Reporting Summary linked to this article.

## Supplementary information

Supplementary Information

Reporting Summary

## Data Availability

Structural data are available via the Protein Data Bank (PDB 7B5P) and the Electron Microscopy Data Bank (EMDB-12043). Any remaining information can be obtained from the corresponding author upon reasonable request
