# Author Correction: Cycloalkane-modified amphiphilic polymers provide direct extraction of membrane proteins for CryoEM analysis

**DOI:** 10.1038/s42003-021-02997-z

**Published:** 2022-01-05

**Authors:** Anna J. Higgins, Alex J. Flynn, Anaïs Marconnet, Laura J. Musgrove, Vincent L. G. Postis, Jonathan D. Lippiat, Chun-wa Chung, Tom Ceska, Manuela Zoonens, Frank Sobott, Stephen P. Muench

**Affiliations:** 1 School of Biomedical Sciences, Faculty of Biological Sciences & Astbury Centre for Structural and Molecular Biology, University of Leeds, Leeds, LS2 9JT UK; 2 Université de Paris, Laboratoire de Biologie Physico-Chimique des Protéines Membranaires, CNRS, UMR 7099, F-75005 Paris, France; 3 Institut de Biologie Physico-Chimique, Fondation Edmond de Rothschild pour le dévelopement de la recherche scientifique, F-75005 Paris, France; 4 Wellcome Centre for Anti-Infectives Research, Drug Discovery Unit, Division of Biological Chemistry and Drug Discovery, University of Dundee, Dundee, DD1 5EH UK; 5 GlaxoSmithKline, Gunnels Wood Road, Stevenage, SG1 2NY UK; 6 UCB Pharma, Slough, SL1 3WE UK; 7 School of Molecular and Cellular Biology, Faculty of Biological Sciences & Astbury Centre for Structural and Molecular Biology, University of Leeds, Leeds, LS2 9JT UK

**Keywords:** Membranes, Cryoelectron microscopy

Correction to: *Communications Biology* 10.1038/s42003-021-02834-3, published online 25 November 2021.

In the original published version of the Article, Reference^32^ was missing the journal title and the final word of the article title. The reference has been updated in the HTML and PDF versions of the Article to the following:
