# Supplementary material for: Cycloalkane-modified amphiphilic polymers provide direct extraction of membrane proteins for CryoEM analysis
Source: Commun Biol. 2021 Nov 25;4:1337. doi: 10.1038/s42003-021-02834-3 (PMC8617058; doi:10.1038/s42003-021-02834-3)
Supplement: Supplementary file 1 — Supplementary Information [file 42003_2021_2834_MOESM1_ESM.pdf]

# Supplementary Information

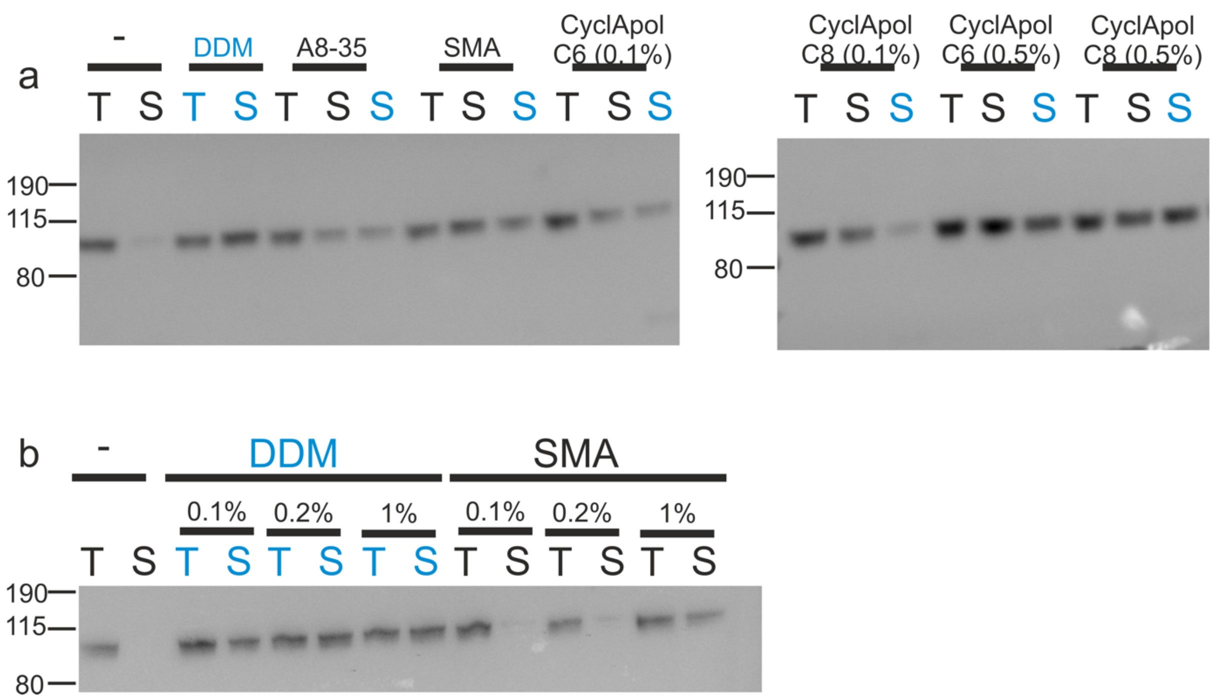


**Figure S1. Solubilisation of AcrB from the *E. coli* membrane with SMA, A8-35 and CyclAPols.** Western blots of AcrB, using anti-His antibody, revealing total (T) and soluble (S) fractions following solubilization of the membrane, at a total membrane protein concentration of 1 mg/mL, for 2 hours. Blue indicates the solubilization is done at 4˚C, all others are at 25˚C. (**a**) The polymer concentrations used in each solubilization condition are as indicated (all (*w/v*)) in the figure. For DDM, the concentration is 1%. The hyphen is a control indicating no solubilizing agent added. The CyclAPols work reasonably well (68-88% solubilisation) at 0.1%, (**a**) and nearly as well as SMA at equal concentration (0.5%), whereas SMA does not function well at lower concentrations (**b**). Additionally, while CyclAPols appear to work poorly at 0.1% and 4˚C, this may be remedied by using higher (0.5%) concentrations.

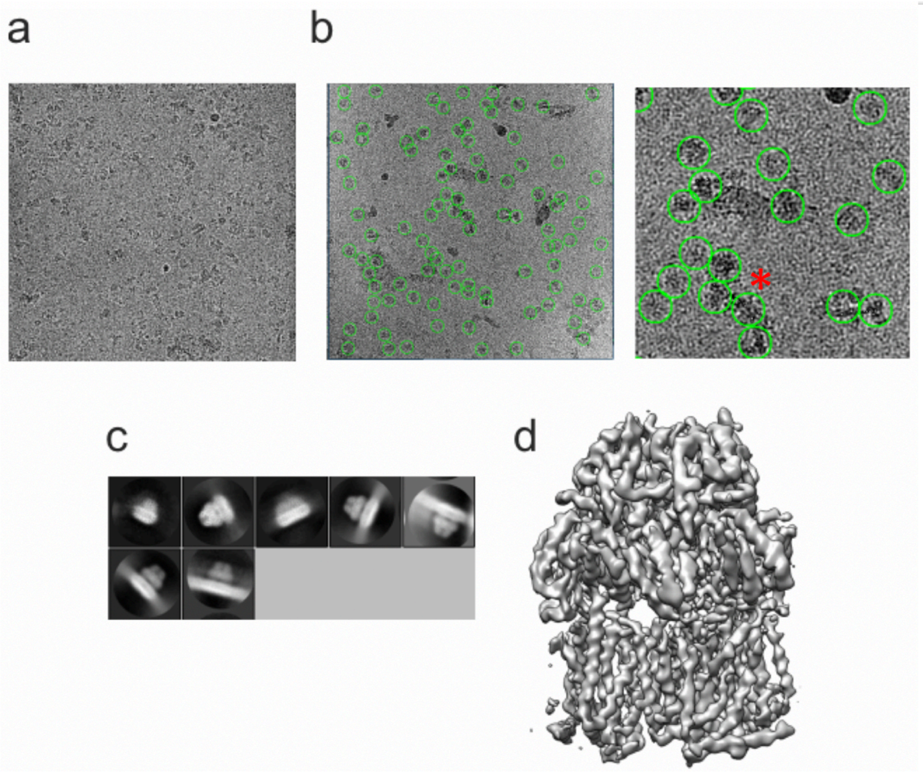


**Figure S2: CryoEM of AcrB in CyclAPol $C_6$-$C_2$-50.** (**a**) Representative micrograph of AcrB purified in $C_8$-$C_0$-50. (**b**) Representative micrograph of AcrB purified in $C_6$-$C_2$-50 (left) with picked particles highlighted by a green circle. (Right) 2x zoom of the micrograph demonstrating despite some aggregation of the protein, it is clear that some trimers are also assembling in near linear chains (red star). (**c**) Selected 2D classes of AcrB in $C_6$-$C_2$-50 following one round of 2D classification. While some classes show a well defined AcrB trimer others show a more diffuse region around the transmembrane domain which may be a consequence of the “strings” of AcrB seen in the raw data (red star). (**d**) Side view of cryoEM map at 4.5 Å final resolution of AcrB solubilized in the $C_6$-$C_2$-50 at threshold of density 0.0235.

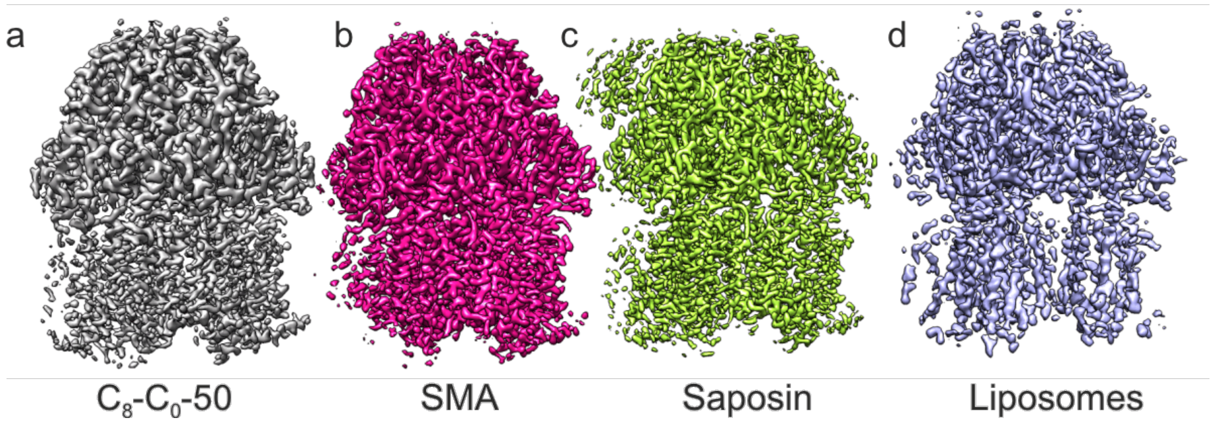


**Figure S3: Comparison of close similarity for AcrB cryoEM structures in various amphipathic environments.** The cryoEM map is shown at similar threshold density for AcrB in (**a**) $C_8$-$C_0$-50 at 3.2 Å resolution [EMD, this study], (**b**) SMA at 3.2 Å resolution [EMD 7074], (**c**) Saposin at 3.27 Å [EMD 10185], and (**d**) Liposomes at 3.9 Å [EMD 22050].

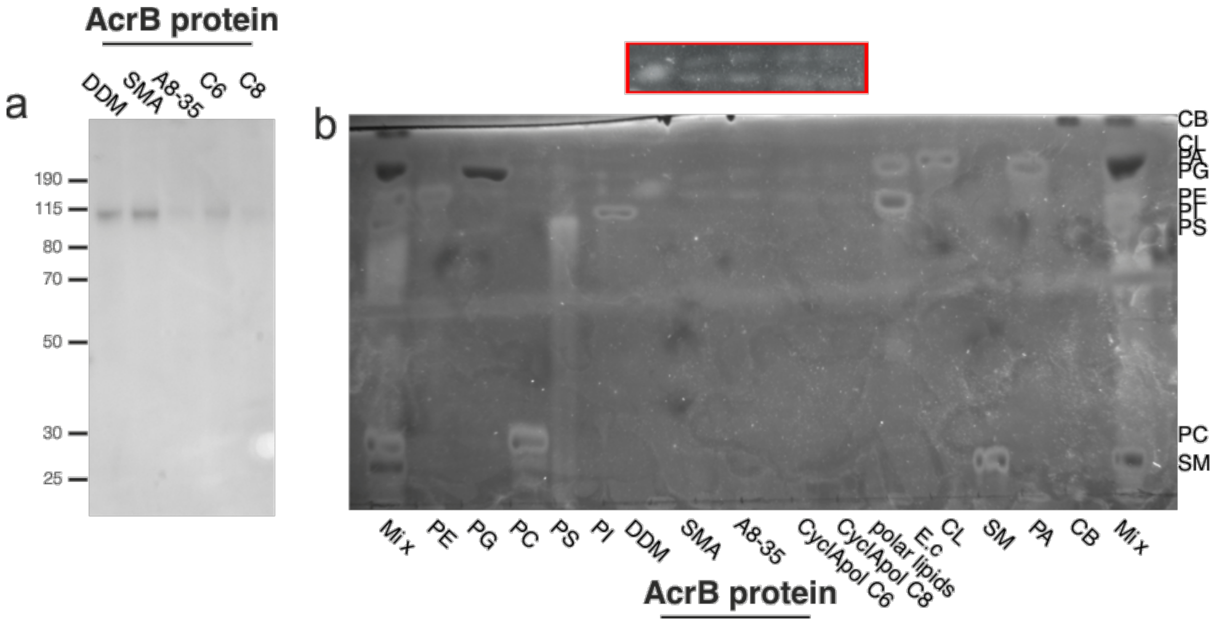


**Figure S4: Thin layer chromatography (TLC) of lipids extracted from purified AcrB.** (**a**) Example SDS -PAGE showing relative amounts of AcrB protein used for lipid extraction. (**b**) example TLC plate showing extracted lipids of purified AcrB solubilised in different environments alongside multiple lipid standards. Inset: lanes of AcrB samples with increased

contrast. This demonstrates similar species and abundance of lipids across AcrB samples despite the differing original amounts of protein, as seen in (**a**).

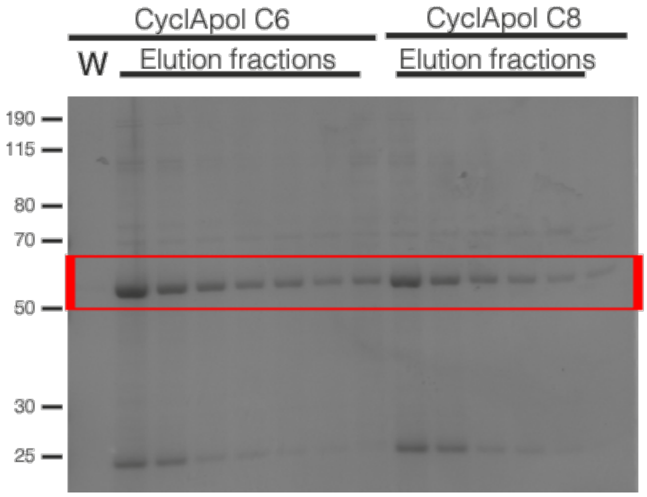


**Figure S5: Example purification of an ABC transporter in CyclAPols.** SDS-PAGE demonstrating purification of a second bacterial target protein outlined in red in $C_6$-$C_2$-50 and $C_8$-$C_0$-50. Purification is similar to that of AcrB (see methods) with solubilisation in a Tris-HCl buffer with CyclAPols at 0.1% and one-step affinity purification with a Strep tag. The difference with AcrB protocol is the solubilization buffer used is 50 mM Tris-HCl pH 8, 250 mM NaCl, 10 % glycerol, with 20 mM and 300 mM imidazole in wash and elution buffers, respectively. Purification is shown with wash (W) and a number of elution fractions. Further studies are ongoing with this target ABC transporter, however this clearly demonstrates the ability of the CyclAPols to purify a second bacterial membrane protein, with high efficiency and purity.

# Supplementary Methods

## Solubilisation assay

*E. coli* AcrB membranes were diluted to 1 mg/mL total protein in solubilisation buffer 20 mM Tris-HCl pH 8, 250 mM NaCl, 5% glycerol. Stocks of DDM (Anatrace) at 25%, A8-35 (Anatrace) at 10%, SMA at 10% and CyclAPols at 5% in dH20 were diluted to indicated final concentration. Samples were incubated with resuspension for 2 hours at room temperature (25˚C) or 4˚C, a 20 µL sample taken (total) followed by ultracentrifugation at 100,000 $xg_{ave}$ for 1 hour at 4ºC, using a TLA 100 rotor. Following centrifugation, supernatant was removed. Equivalent volumes of total and supernatant/soluble samples were mixed with loading buffer and analysed by SDS-PAGE followed by transfer to PVDF membrane using Trans Blot Turbo for western blot analysis. Membranes were blocked in 5% milk in PBS supplemented with 0.1% Tween before incubating for 1 hour with HRP-conjugated anti-His mAb at 1:1000 dilution. The membrane was washed and detected using ECL Chemiluminescence reagent on a G:Box.. Solubilisation estimates are by densitometry of the Western Blot using ImageJ and Soluble/Total for each sample.

## Lipid extraction

Lipids from a 200 µL protein sample were extracted by a modified Bligh and Dyer method[1,2]. Briefly, 160 µL ice-cold chloroform (CHCl3) and 320 µL of ice-cold of methanol containing BHT (50 µg/mL) were added to sample with occasional vortex mixing for 20 min, followed by 150 µL of water, at which point a biphasic mixture was formed. The sample was centrifuged for 5 min at 2,000 xg and the upper (aqueous) phase removed and retained. A second extraction step was performed on the upper aqueous phase and both organic phases were combined and washed with 150 µL $H_2O$ as above. The organic phase was dried under stream of nitrogen and resuspended in 1:1 chloroform:methanol.

Lipid standards from Natural Phospholipids kit (Larodan) or individually from Avanti are 2 mg/mL in 100% chloroform and stored at -20C until use.

HPTLC was performed on HPTLC silica plates (LiChorsphere Silicagel 60) as described previously[3,4]. In short, plates were dried at 100 °C for 20 min, then washed with methanol/ethylacetate (6/4), and heated to 110 °C for 30 min. Samples were applied 1 cm above lower edge of the plate. 3x 2µL of samples and 2µl of each standard are spotted on the plate and placed into a vapour saturated TLC chamber with TLC solution 1 (dichlormethane/ethylacetate/Acetone: 80/16/4). Upon reaching 10 cm plates were removed and air dried, then returned to the saturated TLC chamber with TLC solution 2 (chloroform/ethylactetate/acetone/isopropanol/ethanol/methanol/$H_2O$/acetic acid: 30/6/6/6/18/28/6/2). After air-drying plates were sprayed with a 10% CuSO4/8% phosphoric acid stain and developed for 10 min. at 145 °C. The resulting fluorescent stain was recorded on a G-box system using TAMRA setting.

**Supplementary References**


1. Reis, A. *et al.* A comparison of five lipid extraction solvent systems for lipidomic studies of human LDL. *J. Lipid Res.* **54**, 1812–1824 (2013).
2. Bligh, E.G. and Dyer, W. J. Canadian Journal of Biochemistry and Physiology. *Can. J. Biochem. Physiol.* **37**, (1959).
3. Churchward, M. A., Brandman, D. M., Rogasevskaia, T. & Coorssen, J. R. Copper (II) sulfate charring for high sensitivity on-plate fluorescent detection of lipids and sterols: quantitative analyses of the composition of functional secretory vesicles. *J. Chem. Biol.* **1**, 79–87 (2008).
4. Rohwedder, A., Knipp, S., Roberts, L. D. & Ladbury, J. E. Composition of receptor tyrosine kinase-mediated lipid micro-domains controlled by adaptor protein interaction. *Sci. Rep.* **11**, 1–10 (2021).
